# Protein Fragments: From Biomarkers to Therapeutic Targets—A Focus on α1‐Antitrypsin‐Derived Peptides

**DOI:** 10.1155/mi/6516930

**Published:** 2026-08-31

**Authors:** Friedemann Börner, Tomasz Iwanicki, Julia Held, Joanna Chorostowska-Wynimko, Aleksandra Jezela- Stanek, Sabina Janciauskiene

**Affiliations:** ^1^ Institute of Clinical Chemistry and Laboratory Diagnostics, Jena University Hospital, Jena, Germany, uniklinikum-jena.de; ^2^ Department of Biochemistry and Medical Genetics, School of Health Sciences in Katowice, Medical University of Silesia, Katowice, Poland, sum.edu.pl; ^3^ Department of Respiratory Medicine, Member of the German Center for Lung Research (DZL), Biomedical Research in Endstage and Obstructive Lung Disease Hannover (BREATH), Hannover Medical School, Hannover, Germany, mh-hannover.de; ^4^ Department of Genetics and Clinical Immunology, National Institute of Tuberculosis and Lung Diseases, Warsaw, Poland

**Keywords:** biological activities, biomarkers, diseases, inflammation, protein fragments, proteolysis, *SERPINA1* gene

## Abstract

Protein fragments are increasingly recognized as both biomarkers and therapeutic targets, offering important insights into disease mechanisms and potential intervention strategies. Evidence from in vitro and in vivo studies indicates that these fragments are not merely degradation products but possess distinct biological activities, actively modulating immune signaling and contributing to both acute and chronic inflammatory processes. Here, we provide an overview of proteases and their inhibitors, with a particular focus on peptide fragments generated through proteolytic cleavage. Special emphasis is placed on fragments derived from α1‐antitrypsin (AAT), an acute‐phase glycoprotein and major inhibitor of neutrophil elastase and other serine proteases. AAT‐derived peptides of varying lengths, generated by both target and nontarget proteases, including metalloproteases, have been detected in human biological fluids and tissues. Beyond reflecting proteolytic activity, these peptides provide clinically relevant information on disease‐associated inflammation and tissue remodeling. Accordingly, they are emerging as promising diagnostic, monitoring, and predictive biomarkers. Here, we summarize current knowledge on cleaved AAT fragments, their biological functions, and their potential clinical applications.

**Summary**

• Proteolytic cleavage of full‐length proteins generates bioactive peptides.
• Peptide profiles reflect protease activity and inflammatory processes.
• AAT‐derived peptides arise from target and nontarget protease cleavage of AAT.
• AAT peptides are emerging as markers of various disease activity and progression.

## 1. Introduction

### 1.1. Proteases, Their Specific Inhibitors, and the Physiological and Pathological Implications of Protein Cleavage and Peptide Fragment Generation

#### 1.1.1. Proteases

Proteases constitute ~2% of all known human proteins and are involved in key physiological functions, including immune responses, coagulation, tissue remodeling, and cell death [1]. Proteases are traditionally classified into five major groups based on their catalytic mechanisms: serine, cysteine, aspartic, metallo‐, and threonine proteases.

**Figure 1:**
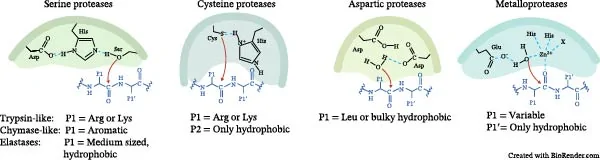
Schematic representation of the proteolytic activity and catalytic mechanisms of the major classes of proteases.

Proteases are specialized enzymes that catalyze peptide bond hydrolysis, thereby regulating protein turnover, processing, and function. Proteolytic cleavage is a tightly controlled, structure‐dependent process that relies on specific interactions between enzymes and their substrates. Consequently, susceptibility to proteolysis is influenced by protein conformation, accessibility of cleavage sites, and post‐translational modifications, including phosphorylation, glycosylation, and ubiquitination [2, 3]. Beyond these structural determinants, proteases often exhibit distinct amino acid sequence preferences that further govern substrate recognition and cleavage‐site specificity (Figure 1).

Proteases are commonly classified according to the catalytic residue or cofactor present in their active site. Serine proteases, including trypsin, chymotrypsin, elastase, and thrombin, utilize an active‐site serine residue to hydrolyze peptide bonds and exhibit characteristic substrate preferences that determine cleavage specificity [4, 5]. Cysteine proteases, such as caspases, cathepsins, and papain, rely on an active‐site cysteine residue and often recognize specific sequence motifs surrounding the scissile bond [6, 7]. Metalloproteases, including matrix metalloproteinases (MMPs) and members of the ADAM family, require divalent metal ions, typically Zn^2+^, for catalytic activity and frequently exhibit preferences for residues adjacent to the cleavage site [8–10]. Threonine proteases are primarily represented by the catalytic core of the multi‐subunit proteasome system [11–13]. Under physiological conditions, proteolysis is tightly regulated and essential for maintaining tissue homeostasis, as well as for coagulation, tissue remodeling, and immune regulation. In contrast, dysregulated proteolytic activity contributes to the pathogenesis of a wide range of diseases, including chronic inflammatory disorders, cancer, neurodegenerative diseases, and coagulopathies.

#### 1.1.2. Endogenous Protease Inhibitors

To preserve tissue homeostasis and prevent uncontrolled proteolysis, protease activity is regulated by endogenous inhibitors, including serpins, cystatins, and tissue inhibitors of metalloproteinases (TIMPs) [14]. These inhibitors are themselves susceptible to proteolytic cleavage by both target and nontarget proteases, a process that can alter their inhibitory activity and consequently influence the local protease–antiprotease balance. Among these regulatory proteins, serpins (serine protease inhibitors) employ a unique suicide‐substrate mechanism in which proteolytic cleavage within the reactive center loop (RCL) triggers a substantial conformational rearrangement that traps the target protease in a stable covalent complex, thereby irreversibly inactivating both the enzyme and the inhibitor [15, 16]. A well‐known example is α‐1 antitrypsin (AAT), which neutralizes neutrophil elastase through this mechanism, resulting in the formation of an irreversible AAT–elastase complex and the functional inactivation of both molecules [17].

Whereas cleavage of serpins at their RCL results in protease inhibition through stable complex formation, cleavage at nonfunctional sites or by noncognate proteases generally disrupts the inhibitor structure without conferring inhibitory activity. Consequently, the inhibitor function is lost, allowing increased proteolytic activity. A comparable mechanism exists in the MMP system, where proteolytic degradation of TIMPs can release the MMP activity and promote extracellular matrix remodeling. These processes are particularly important in pathological conditions such as chronic inflammation and cancer, where disruption of the protease–antiprotease balance can drive tissue injury, invasion, and disease progression [18, 19].

Proteolytic processing of endogenous inhibitors represents a dynamic regulatory mechanism that fine‐tunes protease activity in response to physiological demands such as inflammation, tissue repair, and apoptosis. In addition, cleavage events can generate bioactive peptide fragments that modulate cell signaling, immune responses, and tissue homeostasis, adding an additional layer of complexity to protease‐regulated networks [20]. These fragments may act locally or systemically by engaging cell surface receptors, altering signaling pathways, or modulating immune cell behavior, adding an additional layer of regulation to protease‐driven processes.

#### 1.1.3. Proteolytic Fragments and Their Biological Roles

Beyond protein degradation, proteolytic processing can generate bioactive fragments with distinct biological functions. In many biological systems, proteolysis serves as an essential mechanism that converts inactive precursors into functionally active molecules. A classic example is the conversion of proinsulin into active insulin, which is required for glucose homeostasis [21] or pro‐interleukin‐1β (pro‐IL‐1β) requires proteolytic cleavage by caspase‐1 to generate mature biologically active IL‐1β, a central cytokine in inflammatory signaling [22]. In immune regulation, proteolytic cascades generate key effector molecules. For example, complement activation involves the sequential cleavage of C3 and C5, yielding the anaphylatoxins C3a and C5a, which orchestrate inflammatory and chemotactic responses [23]. In parallel, proteases such as cathepsins and granzymes contribute to host defense by processing pathogen‐derived proteins and facilitating immune effector mechanisms that promote pathogen clearance [24]. Collectively, these proteolytic systems form an interconnected network that not only drives innate immune activation but also shapes the magnitude and specificity of immune responses.

Proteolysis also plays a role in tissue remodeling, where MMPs degrade extracellular matrix components to regulate wound healing, angiogenesis, and fibrosis [25]. However, dysregulated proteolytic activity can drive pathological remodeling and disease progression. In cancer biology, proteolytic systems contribute to tumor progression through multiple mechanisms, including the degradation of key regulatory proteins such as p53 and retinoblastoma protein, thereby disrupting cell cycle control and apoptosis [26, 27]. These examples highlight proteolysis as a fundamental regulatory mechanism that not only degrades proteins but also generates biologically active fragments that shape physiological and pathological processes.

Proteolysis‐derived fragments are increasingly recognized as clinically relevant molecular entities across a broad spectrum of diseases (Table 1). In Alzheimer’s disease, sequential cleavage of amyloid precursor protein (APP) by β‐secretase (BACE1) and γ‐secretase generates amyloid‐β peptides (Aβ40 and Aβ42), which serve as established biomarkers for early diagnosis and disease progression [28–30]. In coagulation disorders, fibrin degradation products such as D‐dimer reflect ongoing fibrinolysis and are widely used as a rule‐out test for thrombosis and pulmonary embolism [31]. Similarly, circulating proteolytic fragments derived from AAT, together with procalcitonin‐derived peptides, have been associated with sepsis severity and hold promise as prognostic biomarkers [32, 33]. In cardiovascular disease, N‐terminal pro‐brain natriuretic peptide (NT‐proBNP) serves as a robust indicator of heart failure severity and prognosis [34, 35]. Beyond these soluble peptide markers, ubiquitin fragments reflect impaired proteasomal degradation and are implicated in both cancer and neurodegenerative disorders [36]. In musculoskeletal and fibrotic diseases, MMPs‐generated collagen fragments serve as indicators of extracellular matrix turnover and tissue degradation [37]. Furthermore, in the ECLIPSE COPD cohort, specific elastin degradation products were associated with a poor prognosis [38]. Collectively, these examples illustrate that protease‐generated fragments represent a heterogeneous class of disease‐associated molecules with significant diagnostic and prognostic value [39].

**Table 1:** Examples of disease‐specific protein fragments.

Protein fragment	Associated disease	Clinical significance	References
Amyloid‐beta (Aβ42, Aβ40)	Alzheimer’s disease	Early diagnosis, progression marker	[30]
Fibrin degradation products (D‐dimer)	Thrombosis, pulmonary embolism	Rule‐out test for clotting disorders	[31]
AAT peptide, C42; Procalcitonin fragments	Sepsis monitoring	Potential prognostic markers	[32, 33]
NT‐proBNP (N‐terminal pro‐brain natriuretic peptide)	Heart failure	Severity and prognosis indicator	[34, 35]
Ubiquitin fragments	Cancer, neurodegeneration	Reflects proteasome dysfunction	[36]
MMP‐generated collagen fragments	Arthritis, fibrosis	Tissue degradation marker	[37]
Elastin fragments	COPD	Potential prognostic markers	[38]

## 2. AAT as a Precursor for Proteolytically Generated Peptides

The 12.2 kb *SERPINA1* gene, encoding AAT, located on the long arm of chromosome 14 at position 32.13 (14q32.1) [40], consists of three noncoding regions (1a–c), four protein‐coding exons (II–V), and six introns [41]. To date, more than 200 *SERPINA1* variants have been identified, many of which result in quantitative and/or qualitative alterations of AAT, an acute‐phase glycoprotein encoded by this gene [42]. Native AAT is synthesized as a 418‐amino‐acid precursor containing a 24‐amino‐acid N‐terminal signal peptide, which is cleaved during secretion to yield a mature 394‐amino‐acid protein. The mature protein is glycosylated at three asparagine residues (Asn46, Asn83, and Asn247), resulting in a glycoprotein with an approximate molecular mass of 52 kDa [43]. AAT is one of the most abundant circulating proteins after albumin and immunoglobulins, with a normal serum concentration of 1.3–2 g/L. The liver is the principal source of AAT (~80%–90%), although extrahepatic production by epithelial cells, alveolar cells, tissue macrophages, monocytes, and granulocytes also contributes to the systemic and local AAT pool [44].

Physiologically, AAT is a key regulator of the protease–antiprotease balance, preserving extracellular matrix integrity, particularly elastin, by inhibiting neutrophil‐derived serine proteases such as neutrophil elastase, proteinase 3, and cathepsin G, which are released during inflammatory responses [45]. Beyond serine proteases, AAT has also been reported to modulate broader proteolytic networks by inhibiting selected cysteine proteases, including calpains and caspases‐1 and ‐3, as well as certain MMPs, such as gelatinase B (MMP‐9), thereby contributing to tissue protection and the resolution of inflammation [46, 47]. These activities position AAT as a central regulator of tissue homeostasis, repair, and regeneration.

Deficiency of functional circulating AAT, most commonly resulting from inherited *SERPINA1* variants, leads to AAT deficiency (AATD), a condition strongly associated with increased susceptibility to chronic lung and liver diseases [48]. In addition to genetic variation, AAT can undergo post‐translational modification, including polymerization, oxidation, proteolytic cleavage, and interactions with other biomolecules, all of which can significantly alter its structure and function [49]. For example, polymerized AAT accumulates predominantly within hepatocytes, where it can induce chronic cellular stress and inflammation, ultimately contributing to progressive liver disease, including fibrosis, cirrhosis, and, in severe cases, hepatocellular carcinoma [50]. In contrast, oxidative modifications, particularly at critical methionine residues, impair AAT antiprotease activity and reduce its protective capacity in inflamed tissues, such as the lung [42]. Proteolytic cleavage, while abolishing the canonical inhibitory function of AAT, generates AAT‐protease complexes that have been reported to enhance *SERPINA1* gene expression in hepatocytes, thereby promoting compensatory synthesis of functional AAT [51]. Importantly, these post‐translational modifications not only result in an acquired functional deficiency of native AAT but also generate structurally modified or cleaved/fragmented AAT molecules with potentially distinct biological activities [52, 51].

## 3. Generation of AAT Fragments

AAT is regulated not only at the level of expression and post‐translational modification but also undergoes proteolytic processing, generating a range of biologically relevant fragments. The best‐characterized form of AAT fragmentation occurs during its canonical inhibitory interaction with target serine proteases. Structurally, AAT adopts a conserved serpin fold comprising a RCL and major β‐sheet structures, including β‐sheet A and β‐sheet C. The C‐terminal region, which contributes to β‐sheet C, is critical for maintaining the structural stability of the molecule. Consequently, mutations or structural perturbations in this region can significantly affect AAT folding, secretion, and functional activity, including its inhibitory capacity and interactions with target proteases and cellular receptors [53].

**Figure 2:**
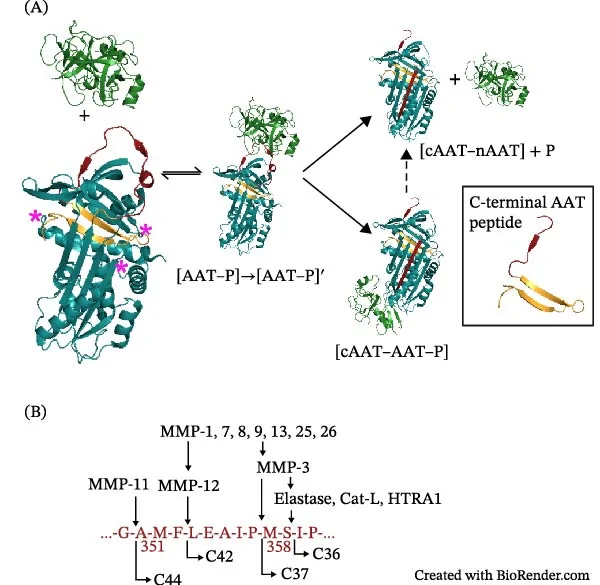
Schematic representation of α1‐antitrypsin (AAT)‐mediated protease inhibition. (A) The reactive center loop (RCL; red) of functional AAT (glycosylation sites indicated by purple asterisks) interacts with target proteases (green) to form an intermediate Michaelis complex ([AAT–P]). Depending on the protease involved, cleavage within the C‐terminal region of AAT results either in the formation of a conformationally stabilized inhibitory complex (nAAT–P) or in dissociation of the protease from cleaved AAT, yielding a noncovalent complex consisting of a small C‐terminal peptide (cAAT) and an N‐terminal fragment (nAAT). (B) Expanded view of the reactive center loop showing protease‐specific scissile bonds targeted by different proteases. Cleavage at these sites generates C‐terminal AAT fragments of varying lengths, designated according to the number of amino acids present in the C‐terminal peptide. Cleavage of the canonical AAT target site (Met358–Ser359) by neutrophil elastase generates the 36‐amino‐acid C‐terminal peptide, C36. Structural models of AAT and AAT–protease complexes were adapted from the Protein Data Bank (PDB; RCSB Protein Data Bank; https://www.rcsb.org accessed 28 January 2026; PDB IDs: 1EZX and 1OPH) and modified using PyMOL (version 2.5.2, Schrödinger). Cat‐L, cathepsin L; HTRA1, high‐temperature requirement serine protease A1; MMP1, interstitial collagenase (fibroblast collagenase); MMP3, stromelysin‐1; MMP7, matrilysin; MMP8, collagenase‐2; MMP9, gelatinase B; MMP11, stromelysin‐3; MMP12, macrophage elastase; MMP13, collagenase‐3; MMP26, matrilysin‐2.

Upon the formation of a stable covalent complex with enzymes such as neutrophil elastase, the RCL of AAT is cleaved at the Met358–Ser359 bond, resulting in a major conformational rearrangement of the molecule (Figure 2A). This conformational transition generates a C‐terminal fragment of ~36 amino acids, which initially remains noncovalently associated with the larger N‐terminal portion before dissociation and potential release into the extracellular milieu or circulation [54]. In addition to this physiological “suicide inhibitor” mechanism, AAT is highly susceptible to noncanonical proteolytic cleavage by a broad spectrum of host‐ and pathogen‐derived proteinases. These enzymes cleave AAT at multiple sites, predominantly within or adjacent to the RCL, leading to the loss of antiprotease activity and generation of heterogeneous peptide fragments without stable complex formation [49, 55] (Figure 2B). Proteases implicated in this process include host enzymes such as cathepsin L, collagenases, stromelysin, gelatinase B (MMP‐9), and collagenase 3 (MMP‐13), as well as bacterial proteases derived from *Staphylococcus aureus*, *Serratia marcescens*, and *Pseudomonas aeruginosa elastase* [56, 57]. In addition, internalized AAT can be cleaved by intracellular caspase‐3 [58, 59]. Conversely, newly synthesized, intracellularly polymerized Z‐AAT, which carries the pathogenic p.Glu366Lys mutation (E366K; unifying the historical Glu342Lys/E342K numbering), undergoes degradation by proteasomal threonine proteases [60]. Following proteasomal processing, certain bioactive fragments exceeding 20 amino acids are actively trafficked out of the cell [61]. These mechanisms highlight intracellular proteolysis as a plausible, yet uncharacterized, source of bioactive, endogenous AAT‐derived peptides.

AAT‐derived peptides have been detected in various human tissues and biological fluids, including urine [62], bronchoalveolar lavage fluid (BALF) [63, 64], gingival crevicular fluid [65], spleen and bile [66], lung tissue [67], carotid arteries [68], the placenta [69, 70], and nipple aspiration fluids [71]. Their widespread distribution reflects continuous protease‐driven turnover of AAT under both physiological conditions and pathological states characterized by inflammation, oxidative stress, and an increased proteolytic burden.

## 4. Biological Roles of AAT Peptides

**Figure 3:**
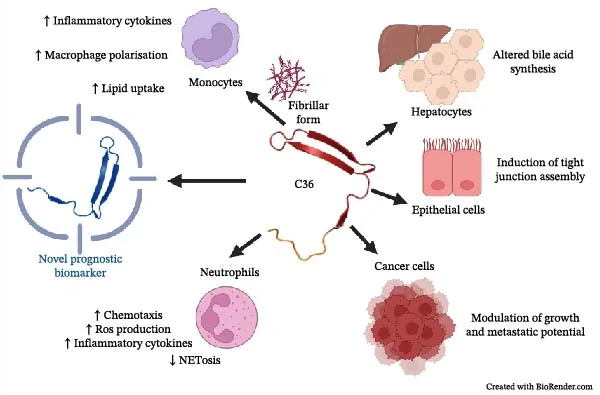
Known cellular functions of the C‐terminal (C36) peptide of AAT. Increasing evidence indicates that C‐terminal AAT peptides, particularly C36, modulate a range of cellular processes and biological responses. These peptides influence innate immune cell function, regulate inflammatory signaling pathways, and contribute to systemic inflammatory responses. In addition, C‐terminal AAT peptides have emerged as promising biomarkers for the early diagnosis, prognostication, and risk stratification of patients with sepsis. ROS, reactive oxygen species; NETosis, neutrophil extracellular trap formation.

**Figure 4:**
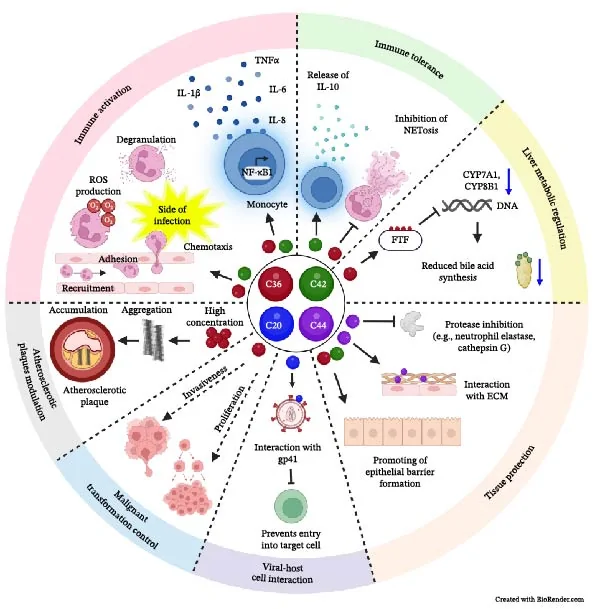
Schematic representation of the biological effects of α1‐antitrypsin (AAT)‐derived peptides on immune and cellular processes. AAT‐derived peptides, including C36 and C42, exert pleiotropic effects on immune and cellular functions. They can promote pro‐inflammatory responses, such as neutrophil activation and increased release of pro‐inflammatory cytokines and chemokines. Concurrently, these peptides also display anti‐inflammatory properties, including C42‐induced IL‐10 production and C36‐mediated inhibition of neutrophil extracellular trap (NET) formation. Beyond immune modulation, AAT‐derived peptides contribute to tissue protection through residual anti‐protease activity, interactions with the extracellular matrix (ECM; e.g., C44), and enhancement of epithelial barrier integrity (C36, C42). In the liver, C36 regulates bile acid synthesis via direct interaction with the farnesoid X receptor (FXR), leading to reduced bile acid production. Additional reported functions include inhibition of HIV infection (C20), modulation of cancer cell invasion and proliferation (C36), and fibrillar structure formation and accumulation within atherosclerotic plaques (C36). Peptides are illustrated as circles: C20 (blue), C36 (red), C42 (green), and C44 (violet).

AAT‐derived peptides exhibit diverse biological activities, modulating both immune and nonimmune cellular processes. Depending on their specific sequence and the local microenvironment, these fragments can elicit either pro‐inflammatory or anti‐inflammatory responses. Among the best‐characterized AAT‐derived peptides, the C36 and C42 fragments predominantly display pro‐inflammatory properties. Both peptides activate neutrophils, promoting their recruitment to sites of inflammation through enhanced chemotaxis, degranulation, ROS production, and endothelial adhesion (Figures 3 and 4) [32, 72]. Additionally, C36 and C42 stimulate the release of pro‐inflammatory cytokines and chemokines, including IL‐1β, IL‐6, TNF‐α, and IL‐8 (Figure 4) [32, 67], whereas C42 also stimulates the release of endothelial adhesion molecules, including sVCAM‐1 and sICAM‐1 [73]. Mechanistically, C36 seems to activate signaling cascades that partially overlap with lipopolysaccharide (LPS)‐induced pathways [67], driving monocyte activation via the NF‐κB, PPARα, PPARγ, and AP‐1 signaling pathways [68].

While C42 primarily promotes leukocyte activation, the C36 fragment has been reported to inhibit neutrophil extracellular trap (NET) formation [74]. This functional divergence highlights the complex and context‐dependent roles of AAT‐derived peptides in fine‐tuning inflammatory responses, not limited to neutrophils.

AAT‐derived peptides also contribute to tissue protection and the maintenance of barrier integrity. The C44 peptide retains partial antiprotease activity and has been shown to reversibly inhibit neutrophil elastase and cathepsin G [69]. In addition, C44 can directly interact with extracellular matrix components [75], suggesting a potential role in preserving tissue architecture during inflammation. Concurrently, both C36 and C42 promote tight‐junction assembly via G‐protein‐dependent signaling pathways, thereby protecting against endothelial barrier dysfunction in murine models [76].

Beyond these immunomodulatory and structural functions, AAT‐derived peptides can influence metabolic and hepatic pathways. Specifically, the C36 peptide has been shown to suppress bile acid synthesis through a direct interaction with the α1‐fetoprotein transcription factor (FTF, also known as LRH‐1). This interaction prevents FTF from binding to its target DNA consensus sequences, downregulating the expression of the rate‐limiting bile acid synthesis enzymes CYP7A1 and CYP8B1 (Figure 4) [77]. Collectively, these findings indicate that AAT‐derived peptides participate in the regulation of hepatic metabolism, expanding their biological relevance beyond classic inflammatory cascades.

Notably, at higher concentrations, the C36 peptide has been shown to self‐assemble into fibrillar structures, which can modulate cellular metabolism, particularly in monocytes [78]. Similar fibrillar forms of AAT‐derived peptides have been identified in atherosclerotic plaques, where they are associated with enhanced receptor‐mediated low‐density lipoprotein uptake (Figure 4). These observations have led to the hypothesis that aggregated AAT peptides may contribute to lipid accumulation and inflammatory processes in atherosclerosis [79].

In conclusion, AAT‐derived fragments constitute a dynamic regulatory network that modulates both the initiation and resolution phases of inflammation.

## 5. Clinical Relevance of the AAT Fragments

AAT‐derived peptides have emerged as bioactive molecules. Through their involvement in inflammatory, immune, and tissue‐remodeling processes, they became attractive as candidate biomarkers for disease diagnosis, prognosis, risk stratification, and monitoring of therapeutic responses. In parallel, preclinical studies and early clinical investigations suggest that selected AAT‐derived peptides may also exhibit therapeutic properties, particularly in inflammatory and immune‐mediated disorders. To date, several AAT‐derived peptides have been evaluated as biomarkers in clinical settings across a range of diseases, including glomerular kidney disease, sepsis, pulmonary fibrosis, gingivitis, carotid artery stenosis, and lung cancer (Table 2).

**Table 2:** Summary table of possible clinical implications of C‐terminal peptides of AAT.

Article authors	Major pathology/disorders	Approach	Key findings	Conclusions
Navarro‐Munoz et al. [62]	Glomerular Kidney Disease (GKD)	Urine MALDI‐TOF MS	Differential expression of UMOD and A1AT peptides	Urinary peptide profiling may allow distinguish GKD noninvasively, particularly its nonproliferative forms.
Tirone et al. [64]	Bronchopulmonary dysplasia (BPD)	Bronchoalveolar lavage fluid/mass spectrometry.	Higher values of specific AAT fragments in severe BPD group.	AAT fragmentation appears to play a central role in lung injury and severe forms of BPD in preterm infants.
Carleo et al. [63]	Idiopathic pulmonary fibrosis (IPF)	Proteomic analysis of bronchoalveolar lavage fluid from IPF patients (stable disease vs acute exacerbation [AE]).	Differentially abundant proteins involved in β‐catenin WNT signaling and carcinogenesis in AE IPF.	Protease/antiprotease imbalance and specific signaling pathways may potentially play a key role in IPF patients experiencing acute exacerbations.
Preiano et al. [65]	Periodontitis, Gingivitis	Gingival crevicular fluid (GCF)/MALDI‐TOF MS	Identification of specific peptides, including AAT C‐36, as biomarkers of gingivitis.	A peptide signature in GCF was identified as a putative biomarker for the diagnosis of gingivitis.
Johansson et al. [66]	Intrahepatic cholestasis and gallstone formation.	Exclusion chromatography in organic solvents to analyze hydrophobic peptides.	Identification of AAT‐related peptides in human bile, plasma, and spleen.	Peptides found may have regulatory roles in antiproteolytic activity or bile secretion processes.
Subramaniyam et al. [67]	Infection and inflammation	Analysis of the effects of the C‐36 peptide on monocytes and neutrophils.	C‐36 peptide stimulates pro‐inflammatory activity and utilizes signaling pathways similar to LPS.	C‐36 mediates effects through LPS signaling pathways, indicating a novel biological activity.
Dichtl et al. [68]	Atherosclerosis	Analysis of atherosclerotic plaques, effects of C‐36 on peripheral blood monocytes.	C‐36 fragment activates PPARα, PPARγ, NF‐κB, and AP‐1 in monocytes and is present in atherosclerotic plaques.	C‐36 has a novel role in monocyte activation, which may be important in atherogenesis and other inflammatory processes.
Niemann et al. [69]	Various pathological conditions	Investigation of C‐44 (SPAAT) as an inhibitor of various serine proteases.	C44 is a reversible inhibitor of human neutrophil elastase and cathepsin G.	C44 might play a role in vivo in protecting proteins from proteases elevated during pathophysiological conditions.
Zhou et al. [71]	Breast cancer	Analysis of nipple aspiration fluids to identify breast cancer biomarkers.	Identification of AAT C41/42 peptide elevated in breast cancer patients.	Elevated C41/42 AAT fragment may be predictive of poor outcome and function as a tumor‐derived suppressor.
Zhou et al. [47]	HIV‐1 infection	Analysis of the C‐terminus of AAT (C) and C terminus‐truncated AAT (ΔAAT) in viral infection including interactions with lymphocyte through gp41.	AAT and C‐peptide, but not ΔAAT, inhibited HIV‐1 entry by directly interacting with gp41.	AAT may inhibit HIV‐1 entry by directly interacting with gp41 through its C‐terminus
Oda et al. [76]	Epithelial barrier dysfunction	Administration of AAT peptides in a murine intestinal injury model (DSS colitis), investigation of tight junction (TJ) formation	Human and murine peptides C35 to C42 promote TJ formation via G‐protein signaling and prevent endothelial barrier dysfunction in vivo.	Therapeutic application if C‐terminal AAT peptides might reduce TJ disruption in inflammatory diseases.
De Araujo et al. [80]	Polymicrobial sepsis	Application of a specific C‐terminal AAT peptide from cord blood (nNIF) in experimental polymicrobial sepsis.	Treatment with nNIF reduced neutrophil extracellular trap formation, cytokine release, and disease severity, increasing survival of mice.	Along standard of care treatment with endogenous AAT peptides may prevent severe outcome in patients with sepsis.
Shapira et al. [81]	Systemic lupus erythematosus (SLE)	Application of the murine ortholog of peptide C36 (UBE) in an animal model of SLE.	Low dose UBE treatment showed beneficial effects on renal function, autoantibodies and abnormal lymphocyte populations in SLE.	AAT peptides might present novel drug candidates to treat SLE
Toldo et al. [82]	Acute myocardial infarction (AKI)	Application of a synthetic truncated C‐terminal AAT peptide (Sp16) in a model of AKI.	Sp16 treatment reduce infarct size and preserved cardiac systolic unction via activation of LRP1 signaling.	Early application of Sp16 might lead to cardioprotective signaling in AKI patients.
Van Tassell et al. [83]	Myocardial infarction	Phase1/2 clinical study for therapeutic application of Sp16 in patients with ST segment elevation myocardial infacrtion (STEMI).	A single subcutanous dose of Sp16 is well‐tolerated and tend to reduce the inflammatory response and infarction size.	Sp16 could be used to reduce the acute inflammatory response in STEMI.
Jiang et al. [84]	Preeclampsia	Peptidomics of healthy and preeclampsia placenta tissue. Administration of C‐terminal AAT peptide (C14) in vitro and in a zebrafish xenograft model.	C14 is upregulated in preeclampsia placenta and significantly increase trophoblast proliferation and angiogenesis of HUVECs in vitro and in vivo.	C14 is a driver in preeclampsia pathology and might present a novel target to alleviate this condition.
Schneider et al. [85]	Non‐small cell lung cancer (NSCLC)	AAT peptide analysis in plasma of 222 patients with NSCLC at pathological stage I–III	C36, C37, and C42 showed the strongest correlations with full‐length AAT, and their levels were affected by smoking status. C42 emerged as an independent prognostic marker for overall survival.	Advanced mass spectrometry techniques for protein fragment analysis may offer new opportunities for biomarker discovery, improved patient monitoring, and the identification of potential therapeutic targets in NSCLC and other cancers.

Abbreviations: AP‐1, activator protein‐1; ΔAAT, C terminus‐truncated AAT; ECM, extracellular matrix; GCF, gingival crevicular fluid; LPS, lipopolysaccharide; MALDI‐TOF, matrix‐assisted laser desorption/ionization time‐of‐flight mass spectrometry; NF‐κB, nuclear factor kappa B; PPARα, peroxisome proliferator‐activated receptor alpha; PPARγ, peroxisome proliferator‐activated receptor gamma; SPAAT, short peptide from AAT; UMOD, uromodulin.

Importantly, the biological and clinical relevance of individual AAT‐derived peptides is influenced not only by their amino acid sequence and length but also by the local tissue microenvironment in which they are generated. Different tissues and disease states are characterized by distinct protease repertoires and levels of proteolytic activity, resulting in unique patterns of AAT cleavage and the generation of specific peptide profiles. Furthermore, factors such as tissue‐specific protease expression, local inflammatory conditions, interactions with extracellular matrix components, and the presence of other binding partners may further shape the abundance and activity of individual peptides. Consequently, the AAT peptidome may provide valuable insights into local pathological processes, reflecting disease‐specific alterations in protease activity and tissue homeostasis. This context‐dependent generation of AAT‐derived peptides may explain their utility as biomarkers across a broad spectrum of diseases.

### 5.1. C20 Peptide

Proteomic studies reveal that the small 20‐residue C‐terminal fragment of AAT (C20, residues 375–394: IEQNTKSPLFMGKWNPTQK) is abundantly present in the urine of pregnant women suffering from preeclampsia and could be used as a urine fingerprint for differential diagnosis [86–88].

Another specific 20‐residue AAT peptide, known as VIRIP (residues 353–372: LEAIPMSIPPEVKFNKPFVF), has been found to bind to the gp41 fusion peptide of HIV‐1. This interaction prevents the virus from entering target cells, thereby inhibiting HIV‐1 infection. This discovery highlights its potential as a novel therapeutic agent in the fight against HIV‐1 [89, 90].

### 5.2. C36 Peptide

The 36‐residue C‐terminal fragment of AAT, termed C36 (residues 359–394; SIPPEVKFNKPFVFLMIEQNTKSPLFMGKWNPTQK), is one of the best‐characterized AAT‐derived peptides and has been detected in a variety of biological samples associated with inflammatory and chronic diseases (Figure 3). The C36 peptide has been identified in the plasma of patients with sepsis [55], non‐small cell lung cancer (NSCLC) [85], COPD [91], and pediatric patients with severe inherited AAT deficiency [92]. The C36 has also been detected in disease‐relevant local compartments, including atherosclerotic carotid artery plaques [68], BALF from patients with chronic lung diseases [63, 64], and gingival crevicular fluid from individuals with periodontal disease [65]. The presence of C36 in both systemic and tissue‐specific samples suggests that its generation reflects ongoing proteolytic and inflammatory processes, supporting its potential utility as a biomarker across diverse pathological conditions.

Among the clinical applications investigated to date, the strongest evidence for the biomarker potential of C36 comes from studies in sepsis [55]. Sepsis is a life‐threatening syndrome caused by a dysregulated host response to infection and remains a major cause of morbidity and mortality worldwide. Early identification of patients at risk of severe disease is critical for timely intervention and improved outcomes [93]. Recent studies have demonstrated that circulating C36 concentrations correlate with disease severity and clinical outcome in septic patients [33, 54], suggesting that this peptide may serve as a biomarker for disease monitoring, prognostication, and risk stratification. These findings indicate that C36 reflects not only the proteolytic activity but also the magnitude of the host inflammatory response during sepsis. Elevated circulating C36 levels were recently associated with reduced overall survival and progression‐free survival in patients with NSCLC, suggesting that C36 may also serve as a predictor of disease progression and clinical outcome in malignancy [85].

The clinical relevance of C36 is further supported by studies of a naturally occurring truncated C36‐derived peptide identified in cord blood and neonatal plasma, termed neonatal NET‐inhibitory factor (nNIF; residues 366–393) [94]. Unlike the parent C36 peptide, nNIF potently inhibits NET formation (Figure 3). NETosis is a specialized neutrophil defense mechanism involving the release of decondensed chromatin decorated with antimicrobial proteins to trap and neutralize pathogens [95, 96]. While beneficial during infection, excessive NET formation contributes to immunothrombosis, tissue injury, organ dysfunction, and mortality in sepsis. Experimental studies demonstrated that nNIF administration reduced disease severity and improved survival in murine sepsis models, particularly when combined with standard antibiotic therapy [84, 95, 96]. These findings highlight the potential of AAT‐derived peptides not only as biomarkers but also as therapeutic agents, raising the possibility of future theragnostic approaches in which peptide measurements could guide peptide‐based interventions.

In addition to its biomarker and immunomodulatory functions, shorter derivatives of C36 have also attracted interest as drug‐delivery tools. One such fragment, termed 105Y (CSIPPEVKFNKPFVYLI), belongs to a class of cell‐penetrating peptides known as endoporters. 105Y has been shown to facilitate intracellular delivery of small interfering RNA (siRNA) through endocytic uptake and subsequent pH‐dependent trafficking within endosomal and lysosomal compartments [97]. Although primarily investigated as a delivery vehicle rather than a biomarker, these findings further illustrate the diverse biological and translational applications of C36‐derived peptides.

### 5.3. C42 Peptide

C42, also known as CAAP48, is a 42 amino acid peptide including residues 353–394: LEAIPMSIPPEVKFNKPFVFLMIEQNTKSPLFMGKWNPTQK. Like the C36 peptide, this peptide has been shown to activate neutrophils and induce inflammatory responses [32]. A naturally occurring single nucleotide polymorphism (SNP)‐derived variant of CAAP48, termed CAAP47, contains an amino acid substitution (E→D) and exhibits reduced molecular mass (~4.7 kDa). Importantly, CAAP47 shows significantly attenuated pro‐inflammatory activity compared with CAAP48 [73]. This differential activity highlights the functional importance of subtle sequence variations within AAT‐derived peptides and suggests that even minor structural changes can markedly alter their immunological effects.

Clinically, C42 has recently been proposed as a circulating biomarker of systemic inflammation. Elevated levels have been reported in patients with sepsis, supporting its potential role in disease severity assessment and risk stratification [33]. More recently, C42 concentrations have also been investigated in patients with acute respiratory distress syndrome (ARDS), where they were found to correlate with disease severity in both mild‐to‐moderate and severe cases [85]. These findings suggest that C42 may serve as a biomarker reflecting the intensity of systemic and pulmonary inflammation, although further prospective studies in larger cohorts are required to validate its predictive and monitoring value, particularly in combination with established inflammatory markers such as IL‐6.

Beyond its pro‐inflammatory actions, CAAP48 has also been reported to induce both pro‐ and anti‐inflammatory cytokines, including IL‐10, indicating a complex and context‐dependent immunoregulatory profile. This dual activity may contribute to its involvement in the dysregulated host response observed in sepsis and associated organ dysfunction. Together, these findings suggest that C42 and its variants, such as CAAP47, represent functionally distinct peptides with potential value not only as biomarkers of disease severity but also as modulators of inflammatory responses. A better understanding of their differential biological activities may support the development of more refined diagnostic and therapeutic strategies in sepsis and related inflammatory disorders.

### 5.4. C44 Peptide

The 44‐residue C‐terminal fragment of AAT (residues 351–394: MFLEAIPMSIPPEVKFNKPFVFLMIEQNTKSPLFMGKWNPTQK), either named C44 or SPAAT, represents one of the earliest AAT peptides detected in human placenta and during different pathological conditions [69, 70]. Although, C44 represents a proteolytic cleavage product, it retains some of AATs inhibitory effectiveness against serine proteases, thus protecting tissue from proteolytic damage.

Recent studies proved the existence of C44 in the blood of patients with sepsis [55, 98]. Interestingly, while plasma level of other AAT peptides, such as C36, C42, or C40, tend to be lower in sepsis survivors, the concentration of C44 is higher, showed the opposite trend and might indicate its protective effect [98]. This divergence likely occurs because severe systemic deregulation shifts AAT cleavage kinetics toward shorter fragments or because sepsis downregulates MMP‐11, the specific enzyme responsible for generating C44 [55, 98]. Consequently, sustained circulating concentrations of C44 serve as a promising indicator of preserved immune homeostasis and favorable clinical outcomes.

## 6. Concluding Remarks

Protein fragments, generated through enzymatic cleavage, have significant biological and clinical value. These fragments can influence the function, stability, and interactions of the parent protein and affect various cellular processes such as receptor regulation, signaling, and immune responses. They can also serve as markers for disease states as their presence or their concentration often reflects specific physiological or pathological conditions. They are also explored as therapeutic targets, where modulating the production or activity of specific fragments may offer new treatment strategies for various diseases, including inflammatory, metabolic, and neurodegenerative disorders.

Within this conceptual framework, AAT serves as a particularly informative model, integrating genetic variation, protease–antiprotease balance, and disease‐associated proteolytic processing. Emerging evidence highlights the functional and translational relevance of C‐terminal AAT‐derived peptides, particularly C36 and C42, in the pathophysiology of sepsis and chronic lung and liver diseases. These observations illustrate how proteolytic processing of a precursor protein can generate fragments with distinct and context‐dependent biological activities as well as biomarker potential.

Overall, proteolysis‐derived protein fragments extend the functional proteome and connect protease activity to both disease pathogenesis and translational applications.

## Funding

This study was funded by the German Centre of Lung Research (Grant 82DZL002C1). Open Access funding enabled and organized by Projekt DEAL.

## Conflicts of Interest

The authors declare no conflicts of interest.

## Data Availability

The authors have nothing to report.
